# Interactions of Calcium with Chlorogenic and Rosmarinic Acids: An Experimental and Theoretical Approach

**DOI:** 10.3390/ijms21144948

**Published:** 2020-07-13

**Authors:** Estelle Palierse, Cédric Przybylski, Dalil Brouri, Claude Jolivalt, Thibaud Coradin

**Affiliations:** 1 CNRS, Laboratoire de Chimie de la Matière Condensée de Paris (LCMCP), Sorbonne Université, 4 place Jussieu, 75005 Paris, France; estelle.palierse@sorbonne-universite.fr; 2 CNRS, Laboratoire de Réactivité de Surface (LRS), Sorbonne Université, 4 place Jussieu, 75005 Paris, France; dalil.brouri@upmc.fr (D.B.); claude.jolivalt@upmc.fr (C.J.); 3 CNRS, Institut Parisien de Chimie Moléculaire (IPCM), Sorbonne Université, 4 place Jussieu, 75005 Paris, France; cedric.przybylski@upmc.fr

**Keywords:** chlorogenic acid, rosmarinic acid, calcium, antioxidant, Density Functional Theory

## Abstract

Chlorogenic (CA) and rosmarinic (RA) acids are two natural bioactive hydroxycinnamic acids whose antioxidant properties can be modulated by the chelation of metal ions. In this work, the interactions of these two carboxylic phenols with calcium ions and the impact of such interactions on their antioxidant activity were investigated. UV-Vis absorbance, mass spectroscopy and ^1^H and ^13^C liquid NMR were used to identify complexes formed by CA and RA with calcium. Antioxidant activities were measured by the Bois method. Density functional theory (DFT) calculations were performed to evaluate the most stable configurations and correlated with NMR data. Taken together, these data suggest that calcium ions mainly interact with the carboxylate groups of both molecules but that this interaction modifies the reactivity of the catechol groups, especially for RA. These results highlight the complex interplay between metal chelation and antioxidant properties of natural carboxylic phenols.

## 1. Introduction

Phytopharmacology has attracted much scientific attention in the last decades not only because of its use in traditional medicine over three thousand years, mainly as herbal tea but also in modern medicine since a large proportion of known drugs have been initially identified in plants and are currently used as such or as derivatives. Indeed, it is estimated that at least 25% of all modern medicines are derived, either directly or indirectly, from medicinal plants, primarily through the application of modern technology to traditional knowledge [1]. As a sign of this increasing interest, a list of 442 plants is included in the 2013 version of the European Pharmacopoeia for their therapeutic use. However, control quality to ensure safety and efficacy in the production of traditional medicines is difficult, thus precluding from ensuring the efficiency of a drug based on a plant extract. A more recent approach is to focus on the most active molecules from plants. Among them, polyphenols have been demonstrated to possess numerous health benefits such as antioxidation [2,3,4,5], anti-inflammation [6,7] and antimicrobial activity [3,7,8]. 

**Figure 1:**
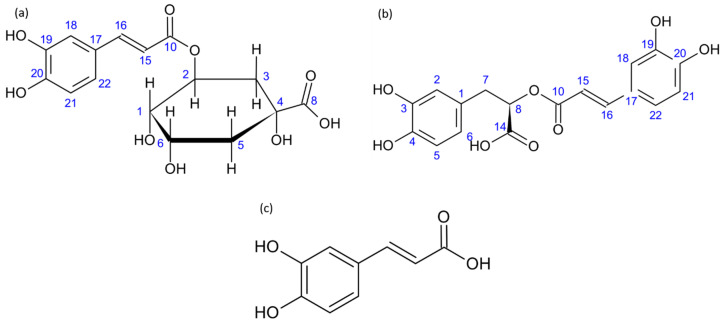
Molecular structure of (**a**) chlorogenic acid, (**b**) rosmarinic acid and (**c**) caffeic acid.

In particular, the most studied compounds include flavonoids, ubiquitous plant secondary metabolites, whose activity is, at least partly, associated with their chelation of transition metal ions [9]. Such a complexation can not only lead to increased solubility, hydrophilicity and bioavailability, but the metal complexes may also possess biological properties different from the ligand alone [9,10,11]. In particular, iron [9,12,13,14] and copper [10,12,15,16,17] playing a key role in living systems, their complexes with flavonoids have been widely studied [18]. Another class of bioactive molecules of vegetal origin with high biological importance is that of carboxylic phenols such as chlorogenic acids [19,20,21], gallic acid [22], rosmarinic acid [23], ferulic acid [24] and ellagic acid [25]. Complexation with transition metal ions was also largely studied, in particular for caffeic acid (Figure 1) derivatives [26,27,28]. In aqueous solutions at neutral pH polyphenolic molecules usually bind to the metal or alkali metal ion by one or more –OH and–COO^−^, if present, mostly resulting in the formation of weak mononuclear complexes [26]. However, so far, their calcium complexes have been poorly characterized. 

In this context, this study aimed at a detailed evaluation of calcium ions interactions with chlorogenic acid (CA) and rosmarinic acid (RA) (Figure 1). UV-Vis absorbance studies were first undertaken to check for the formation of complexes. Electrospray mass spectrometry was then used to determine the stoichiometry of such complexes. The identification of interaction sites was achieved by NMR spectroscopy. A possible structure of calcium complexes was provided with the support of a modeling approach using Density Functional Theory (DFT). Finally, the effect of calcium on the antioxidant activity of both molecules was assessed. A discussion of these results in terms of the most favorable binding site for calcium ions and its influence on CA and RA reactivity, including antioxidant properties, is provided

## 2. Results

### 2.1. Spectroscopic Studies of Calcium Interaction with RA and CA

#### 2.1.1. UV-Visible Spectroscopy

**Figure 2:**
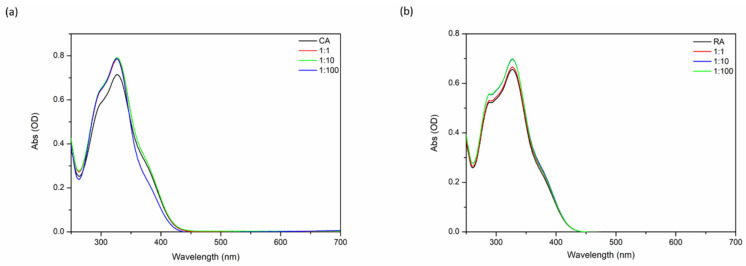
UV-visible spectra of (**a**) chlorogenic acid and (**b**) rosmarinic acid in 1mM (NH_4_)_2_(CO_3_) buffer (pH 7.8) as a function of RA:Ca^2+^ or CA:Ca^2+^ molar ratio (1:1, 1:10, 1:100); RA and CA concentration: 50 µM each one.

Because of its sensitivity to the protonation state of catechol as well as to the influence of the presence of metal ions, UV-vis spectroscopy is a method of choice to study both free phenolic acids and their complexes [29,30,31,32,33]. Figure 2 displays the absorbance spectra of mixtures of RA or CA and calcium chloride at three different RA:Ca^2+^ or CA:Ca^2+^ molar ratios, 1:1, 1:10 and 1:100, recorded in ammonium carbonate buffer (pH 7.8).

In an ammonium carbonate buffer of pH 7.8, the spectrum of RA alone shows a maximum at 325 nm with two shoulders at 288 nm and 380 nm (Figure 2b). Upon calcium addition, no new bands appear but for RA:Ca^2+^ of 1:10 and 1:100 the intensity of the spectrum increased in the 275–375 nm range. For CA alone, the initial spectrum has a similar shape and absorption maximum is observed at 323 nm with two shoulders at 300 nm and 380 nm (Figure 2a). Upon calcium addition, the intensity of the spectrum increases again in the 275–375 nm range. Moreover, for a CA:Ca^2+^ ratio of 1:100, the relative intensity of the shoulder at 380 nm decreases. According to the literature [34], the two bands at 288 nm and 325 nm for RA (and 300 nm and 323 nm for CA) are representative of the fully protonated catechols of the caffeic group of the molecules while the 380 nm band indicates the presence of a deprotonated catechol involving the hydroxyl group on the para position of the caffeic part (Figure 1). In the presence of calcium, no additional band was clearly identified and only minor variations in bands intensity was observed, suggesting that a limited fraction of calcium ions interact with CA and RA and/or that its binding occurs in non-chromophoric groups of the molecules.

#### 2.1.2. Electrospray Mass Spectrometry

Electrospray ionization mass spectrometry (ESI-MS) has previously shown to provide direct evidence for the formation of metal-ligand complexes [10]. The interactions of calcium ions with RA or CA was thus investigated by ESI-MS in positive mode. 

**Figure 3:**
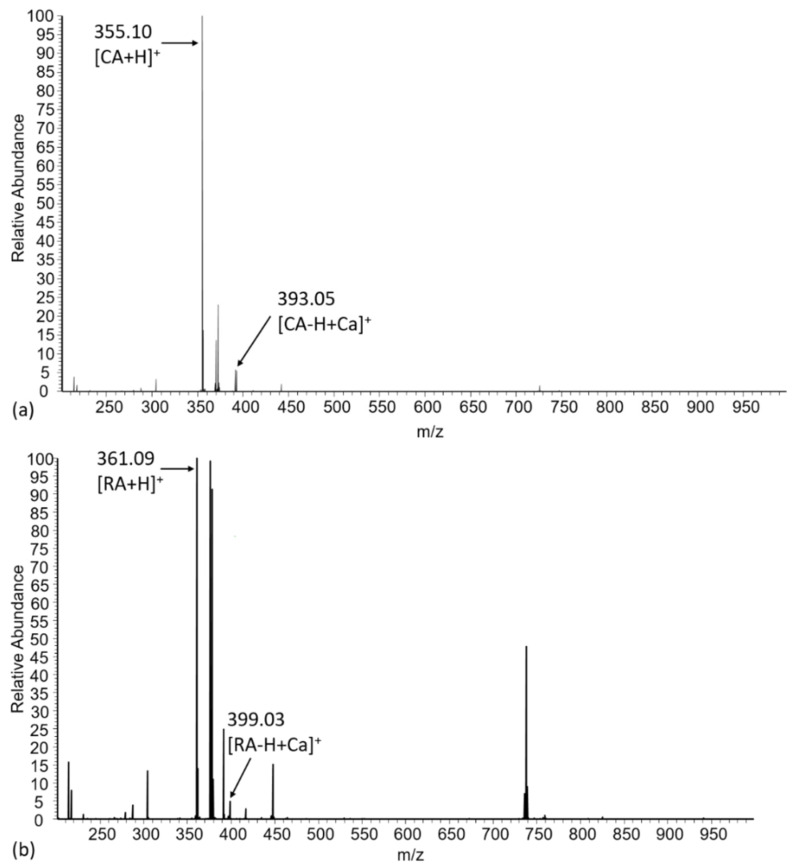
MS spectra of (**a**) chlorogenic acid and (**b**) rosmarinic acid with CA:Ca^2+^ or RA:Ca^2+^ = 1:1 at pH 7.8.

Figure 3 shows the mass spectra of equimolar solutions of RA or CA with Ca^2+^ at pH 7.8 in ammonium carbonate buffer. RA and CA spectra exhibit a major peak corresponding to the protonated molecule, at *m/z* 361.09 ([RA+H]^+^) and 355.10 ([CA+H]^+^), respectively. In addition, [CA−H+Ca]^+^ and [RA-H+Ca]^2+^ species are detected at *m/z* 394.10 and 393.05, respectively, with a significant intensity (≈ 5% of that of the main peak). Additional peaks in Figure 3a,b can be attributed to RA or CA species associated with ammonium. Altogether, interactions of Ca^2+^ with a mono-deprotonated molecule in a 1:1 stoichiometry seem the most favorable at this pH.

#### 2.1.3. NMR Spectroscopy

Because MS data brought to light the interactions of RA and CA with calcium, NMR spectroscopy was performed in an attempt to identify one or several putative complexation sites for calcium ions on RA or CA molecules [14,18,35]. ^1^H and ^13^C NMR spectra of CA and RA without or with calcium were recorded in D_2_O at pH 7 with (RA or CA):Ca^2+^ = 1:100, and corresponding chemical shifts were assigned in accordance with the literature (see Figures S1 and S2) [36,37]. 

**Figure 4:**
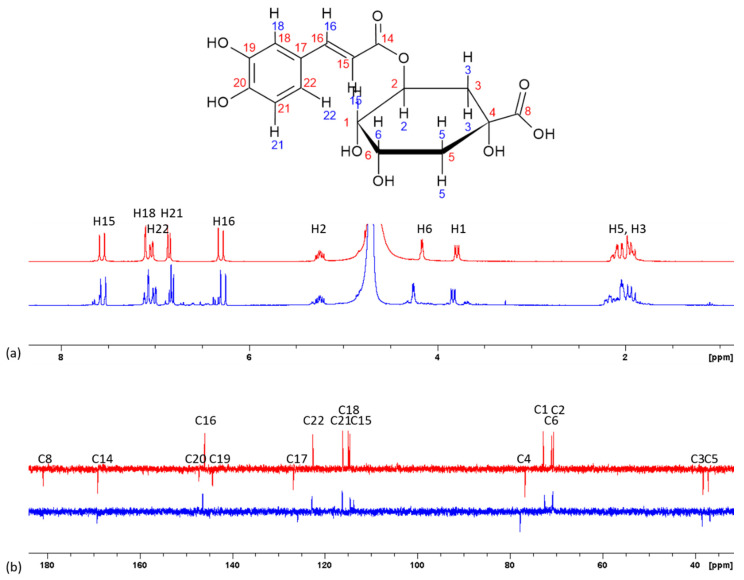
(**a**) ^1^H and (**b**) ^13^C NMR spectra of chlorogenic acid in the absence (red line) and in the presence of calcium, CA:Ca^2+^ = 1:100 (blue line) in D_2_O (pH 7).

For CA, the only protons whose chemical shift varied by more than 0.02 ppm (in absolute value) in the presence of calcium ions were H6, H15, H16 and H21 (Figure 4). Aromatic and vinylic protons were shielded, while H6 from the quinic part of the molecule was deshielded. A complexation of calcium at the carboxylic acid site, leading to an electron-withdrawing effect on the adjacent carbons of the quinic moiety of CA, could explain the shielding of the corresponding protons. However, as signals from H3 and H5 are hardly distinguishable, it is difficult to state about a potential change in their chemical shifts. In parallel, deprotonation/complexation at the catechol site of the molecule could explain the deshielding of aromatic and vinylic protons. Indeed, facilitated deprotonation of one hydroxyl group complexed with calcium would induce a positive mesomeric effect along the conjugated moiety of the molecule.

^13^C NMR analysis shows chemical shift variations up to around 1 ppm (C4 and C17) in the presence of Ca^2+^. Smaller variations (between 0.2 and 1 ppm) were observed for carbons 1, 5, 6, 15 and 16. The mesomeric effect on the conjugative part of the molecule upon catechol deprotonation can lead to the deshielding of carbons in ortho and para positions compared to the deprotonated hydroxyl, and the shielding of meta position. High deshielding of C4 could sign for the complexation near the carboxylate that we can assume to be between the hydroxyl group of C4 and the hydroxyl group of C8 (4-hydroxy-8-hydroxy site). This is consistent with the fact that this site is commonly mentioned as a site of complexation for metals in CA [38]. It is noteworthy that C20 and C19 as well as C8 signals were missing in the presence of calcium. Such modifications could be induced by some relaxation issues and prevent any conclusion about the potential involvement of these positions in complexation. However, from both ^1^H and ^13^C NMR data, it could be hypothesized that calcium complexation could take place either with a carboxylic group or with catechol C19–C20.

**Figure 5:**
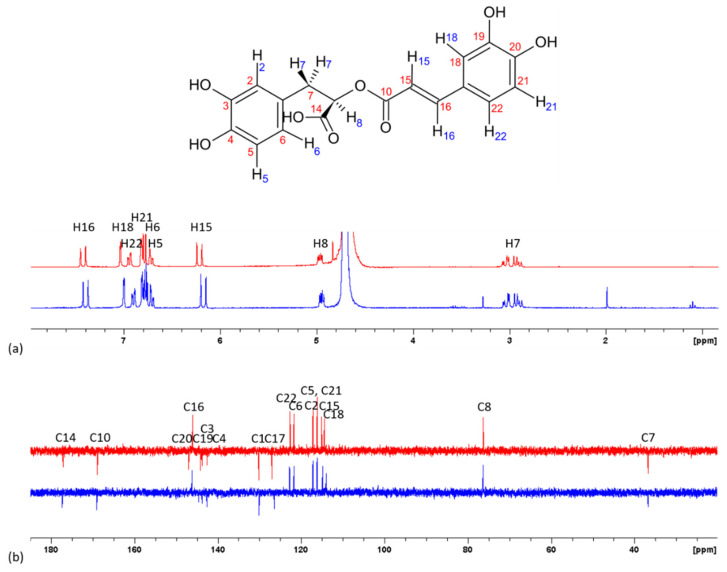
(**a**) ^1^H, and (**b**) ^13^C NMR spectra of rosmarinic acid in the absence (red line) and in the presence of calcium, RA:Ca^2+^ = 1:100 (blue line) in D_2_O (pH 7).

Analysis of the ^1^H spectra of RA showed that protons on C15, C16, C18 and C22 have the largest variation in chemical shift modification upon calcium addition (Figure 5). Complexation would therefore preferably take place on the C19–C20 catechol site. In the ^13^C spectrum, C19 was deshielded (+0.4 ppm), in line with a possible complexation on the catechol site, further confirmed by the observed shielding of C17 and C18 (−0.57 and −0.45 ppm, respectively). This could be explained by a mesomeric effect, that would also impact on the vinylic carbons. Finally, a 0.23 ppm shift of C14 signal was observed, which could be an indication that the carboxylate group is also involved in the complexation, although this hypothesis is not unambiguously confirmed by ^1^H NMR results. 

### 2.2. Density Functional Theory Calculations

**Figure 6:**
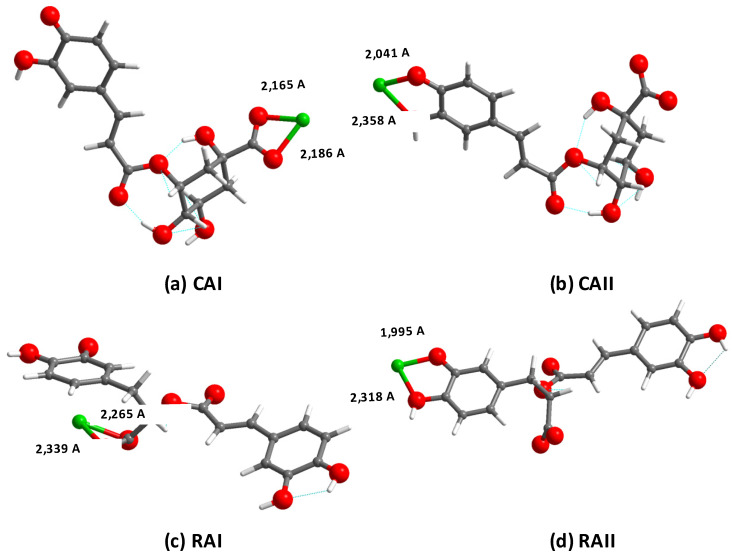
Selected calculated configurations of 1:1 complexes of di-deprotonated (**a**,**b**) CA and (**c**,**d**) RA with one Ca^2+^ ion.

Density functional theory (DFT) calculations were performed for unprotonated forms of RA and CA likely to be relevant at pH 7 on the basis of the pK_a_s of their acidic groups. At pH 7, the carboxylic groups of both compounds are deprotonated since their pK_a_s are 2.92 and 3.90 for RA and CA, respectively. The former value was reported by Danaf et al. [34] and the latter one was calculated using ACDLab software. In order to ensure the neutrality of the 1:1 complex with Ca^2+^, a second deprotonation was assumed to stand at the hydroxyl groups with the lowest pK_a_s, i.e., C20 or C3 hydroxyls for RA whose pK_a_s are 8.36 and 9.56, respectively [34]. Because RA and CA share the same caffeic part, the hydroxyl group on C20 is also assumed to have the lowest pK_a_ value in CA and therefore was chosen as the more likely deprotonation site in this phenolic compound while hydroxyl groups located in its quinic part have pK_a_ representative of aliphatic alcohols, thus above 10. On this basis, two different sites were hypothesized for the calcium complexation via interactions with the oxygen atoms of the carboxylate group or of the catechol group. Optimized structures are shown in Figure 6 and total energies are reported in Table 1. 

Calculated energies indicate that calcium binding to CA at the carboxylate group **(CAI)** leads to a more stable association (−266.07 eV) than at the catechol site **(CAII)** (−265.12 eV). In the case of RA, the calculated energy values show that complexes **RAI** and **RAIII**, i.e., with Ca^2+^–carboxylate interactions, have the lowest energies, namely −270.97 and −269.29 eV. The energies of both complexes formed via Ca^2+^–catechol interactions (**RAII** and **RAIV**) are higher, i.e., −268.86 and −268.58 eV, respectively. 

In addition to energy values, chemical shifts of protons and carbons can be deduced from the modeling using linear calibration curves obtained from some known organic molecules (Figures S3 and S4). For chlorogenic acid alone, a good correlation was observed between experimental and calculated chemical shifts (Figure S5), so that these data were further analyzed for the putative complexes. The most significant chemical shifts variations between free and complexed CA for the configuration **CAI** as obtained by calculation were the same as those observed experimentally, especially the C4 atom (Table S1). Noticeably, for C15, C16 and C20 carbons, calculated chemical shift variations are significant, suggesting that the coordination of calcium atoms at the carboxylate site may impact the conformation and/or hydrogen bond network of the caffeic moiety. For **CAII** configuration, calculated variations in chemical shifts were small and, especially, never larger than the 10 ppm value considered as significant for ^13^C data (Table S2).

Calculated ^1^H and ^13^C chemical shifts of complexes **RAI** to **RAIV** were also compared to the corresponding experimental values (Tables S3–S6). Overall, experimental chemical shift variations between RA alone and in the presence of calcium ions are smaller than for CA, making these data more difficult to interpret. However, a good correlation was again observed between the experimental and calculated chemical shifts of RA alone (Figure S6). Moreover, the most significant changes in the ^13^C chemical shift were calculated for the carbon atoms of the carboxylate group of the **RAI** complex and a catechol one (C20–C21) of **RAIV**, which can be considered as a positive indication of the model accuracy. In the case of **RAI**, several significant changes of calculated values are observed for atoms that are not in the vicinity of the carboxylic complexation site, such as C15, C17, C18 or C22, as previously observed for **CAI**. For **RAIV,** a similar convergence between experimental and calculated variations is also obtained for the C15 group. However, a clear discrepancy is obtained for C16, C19 and C21 for which high variations were calculated but not confirmed experimentally. For instance, for C21, the calculated chemical shift variation is –42.98 ppm while the experimental one is only 0.01 ppm. For **RAII** and **RAIII** complexes, the most significant calculated chemical shift variations upon complexation show a rather poor correspondence with the experimental ones for both ^1^H and ^13^C. 

### 2.3. Effect of Calcium on Antioxidant Activity

**Figure 7:**
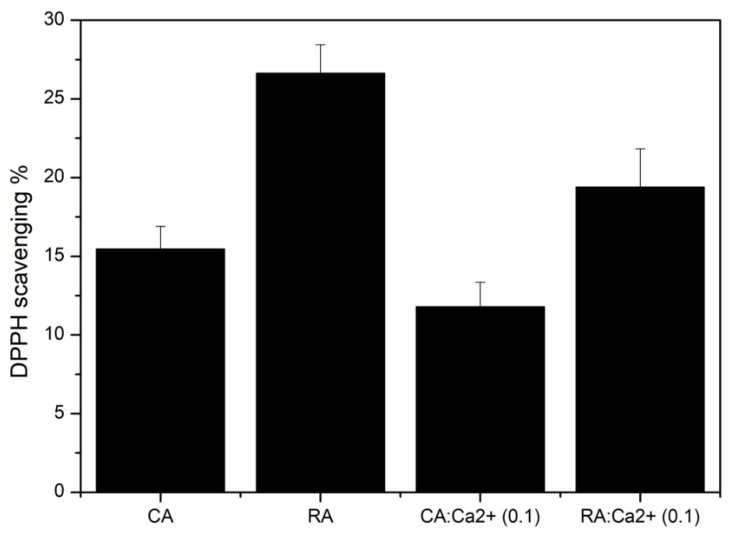
Antioxidant activity of RA or CA alone or in the presence of calcium (molar ratio CA:Ca^2+^ or RA:Ca^2+^ = 1:10). *n* = 3. Error bars correspond to the standard deviation of the three measures.

The effect of calcium chelation on antioxidant activity was assessed according to the DPPH (2,2-diphenyl-1-picrylhydrazyl) radical scavenging method (Figure 7), for 1:10 molar ratio (RA:Ca^2+^ or CA: Ca^2+^). DPPH bleaching percentage was measured after 20 minutes of reaction in the dark. RA is the most effective antioxidant molecule, with a DPPH bleaching percentage of 27.0%, followed by CA (15.4%). The presence of calcium does not affect the antioxidant activity of CA, while the DPPH bleaching percentage decreased from 27.0 to 19.4% for RA. Knowing that phenolic hydroxyl groups are reported to be of particular importance in the antioxidant activity of polyphenols [39], these results would suggest that Ca^2+^ interactions with CA mainly affect its carboxylate group whereas the reactivity of the catechol rings of RA are also sensitive to the presence of calcium.

## 3. Discussion

It has been widely reported that polyphenols exhibit antioxidant and chelation properties that have industrial applications, such as therapeutic agents for the treatment of degenerative diseases or in antimicrobial food packaging [40,41,42,43,44]. These two properties are interrelated as chelation may modulate the oxidative potential of both the coordinating molecule and the coordinated metal [45,46,47,48]. In the case of non-redox active ions, such as alkali metals, only the first effect is expected, which, in turn, may provide an interesting way to study their interactions with the polyphenol ligands.

In the case of chlorogenic acid, two binding sites for calcium are possible: a carboxylate group and a catechol group belonging to the caffeic acid unit. In the case of rosmarinic acid, one carboxylate group and two catechol are putative calcium binding sites. In the literature, the preferential site for Pb^2+^ binding to CA was reported to be the carboxylate group at a low metal:ligand ratio [32], but strong calcium coordination to catechols was often reported for other parent molecules [49]. In the case of RA, no report on non-transition metal divalent cations is available but the binding of alkali metals was found to occur on the carboxylate group [50]. 

At pH 7.8, the carboxylate group of the two acids is deprotonated. However, for both molecules, these groups are not conjugated to the caffeic moiety so that their deprotonation state is expected to have no impact on the UV-vis spectra. Moreover, a weak band associated with partial deprotonation of a catechol group is observed, in agreement with the pK_a_ of ca. 8.4 of the hydroxyl group in the C20 position. Upon calcium addition, only minor variations of the UV-vis spectra were measured, with no significant shift of the absorption maxima, suggesting that only a weak interaction exists between Ca^2+^ and both CA and RA and/or that this interaction occurs at the non-chromogenic carboxylate sites.

In this context, ESI-MS studies do confirm that 1:1 association of calcium with mono-deprotonated CA and RA exist in solution, although they represent only 5% of the ligand alone in equimolar solutions. Accordingly, ^1^H and ^13^C NMR experiments showed only slight variations in chemical shifts, especially for RA, that do not allow to conclude whether calcium interacts with the carboxylate group, catechol rings or both. 

DFT calculation indicates that for both acids, complexation on the carboxylate group leads to the most stable configurations. However, it is important to point out that it was necessary here to consider a deprotonated form of the molecules to ensure the neutrality of the 1:1 complex formed with Ca^2+^, which does not reflect the major protonation state of the two acids at pH 7.8 and can, therefore, explain some of the differences in the amplitude of chemical shift variations between experimental and calculated values. In the case of RA, the choice of the additional deprotonated catechol site has a non-negligible influence on the calculated energies and the correspondence between experimental and calculated NMR chemical shift variations. Nevertheless, important information gained from the theoretical approach is that calcium binding at the carboxylate group can have a marked influence on the caffeic fragment of the molecules, although they are not conjugated, especially for RA.

On the one hand, UV-vis suggests that Ca^2+^ does not significantly interact with the chromophoric caffeic group moiety in neither CA or RA, thus favoring the carboxylate group as the major calcium binding site. On the other hand, NMR studies and antioxidant activity suggest that the catechol groups of RA are influenced by the presence of calcium. The most plausible explanation provided by DFT calculation is that the calcium ion does bind on the carboxylate group but that such a binding modifies the conformation of the aromatic part of the caffeic moiety of RA and therefore the reactivity of its catechol groups. It is important to point out that catechol groups do indeed have a very strong affinity for divalent cations [49], but that, at neutral pH where carboxylate groups are deprotonated while catechols remain unionized, the former can constitute a preferred binding site, as previously shown for Pb^2+^ binding to CA [32].

These results highlight the complexity of the metal-binding mechanisms of chlorogenic and rosmarinic acid and illustrate the sensitivity of their antioxidant properties to such binding, even for non-redox metal ions. They also point out that the identification of the resulting weak complexes is not straightforward and requires the combination of multiple experimental techniques and theoretical approaches.

## 4. Materials and Methods

### 4.1. Chemicals

Chlorogenic acid (CA, min 98%) and rosmarinic acid (RA, min 98%) were purchased from Carbosynth Limited, Compton, United Kingdom. Calcium chloride dihydrate (>99%) was obtained from Jessen Chemicals, Hamburg, Germany. Tris(hydroxymethyl)aminomethane (TRIS, >99.8%), 2,2-diphenyl-1-picrylhydrazyl (DPPH, 97%) and ammonium carbonate ((NH_4_)_2_CO_3_, 99.99%) were purchased from Sigma-Aldrich, Darmstadt, Germany. Deuterium oxide (D_2_O, D 99.9%) and sodium deuteroxide (NaOD, D 99.5%, 40% in D_2_O) were obtained from Eurisotop, Saclay, France.

### 4.2. Spectroscopic Studies

Calcium complexes were prepared in 1mM (NH_4_)2(CO_3_) buffer (pH 7.8) and the same solvent was used to record the spectra. Calcium chloride stock solution was prepared in (NH_4_)_2_(CO_3_) buffer at a concentration of 0.01 M, and RA and CA stock solutions were prepared in DMSO. To prepare the complexes in solution, solutions of CaCl_2_ and RA or CA were mixed and diluted in 1 mM (NH_4_)_2_(CO_3_) buffer to obtain the concentration of interest. UV-visible spectroscopy was performed on a Libra Biochrom S60 spectrometer, between 250 and 600 nm. Electrospray ionization mass spectrometry (ESI-MS) studies were conducted on an LTQ orbitrap mass spectrometer, with a capillary voltage of 20 V and tube lens voltage of 70 V, in positive ion mode. Resonant excitation was carried out using collision-induced dissociation (CID), normalized collision energies (NCE) were between 5 and 30%. All data analysis was performed using Thermo Xcalibur™ software (4.3, Thermo Fischer Scientific, Waltham, MA, USA). Solutions were prepared in ammonium carbonate buffer 1 mM at pH 7.8, from stock solutions of rosmarinic or chlorogenic acid in methanol and above-described stock solution of calcium chloride. Samples were introduced at the rate of 20 µL.min^−1^ with a 500 µL syringe. Complexation of calcium by rosmarinic and chlorogenic acids was studied by ^1^H and ^13^C NMR, on a Bruker 300 MHz spectrometer equipped with a BBFO probe. Rosmarinic acid and chlorogenic acid were dissolved in D_2_O in the presence of calcium chloride with ratio RA:Ca^2+^ or CA:Ca^2+^ = 1:10, and pH was adjusted to 7 with NaOD 0.01 M.

### 4.3. Molecular Modeling 

Total electronic energy calculations were performed with the VASP version 5.4.1 software [51] within the density functional theory, using projected augmented wave functions, with the PBE functional [52] in the generalized gradient approximation and periodic boundary conditions. The Brillouin zone sampling was limited to the gamma point. For this purpose, periodic boxes (30 × 30 × 20 nm) were generated containing the Ca-complexes. Ca-complex models for rosmarinic and chlorogenic acid were built thanks to the MAPS interface [53], which allowed to conveniently prepare and submit DFT calculations, recover and analyze results. The simulations were performed with an energy cutoff of 400 eV for the plane-wave basis set. The self-consistent field cycles were converged to 10^−4^ eV, and atomic relaxations to 5.10^−2^ eV.Å^−1^. The zero damping DFT-D3 method of Grimme [54] was used to take into account the Van der Waals contribution to the total energy. Initial guesses of geometries were chosen on the basis of chemical common senses such as avoiding misleading secondary minima of the potential energy surfaces. Hessian matrices and NMR chemical shifts tensors were computed for the structure’s optimized geometries according to the linear response method [55,56]. The calculated shielding σ constants, defined as the ratio between the induced magnetic field and the applied uniform external magnetic field were used to predict the chemical shifts δ for ^1^H and ^13^C nuclei. Linear correlations between σ and chemical shifts δ were established using a set of organic molecules with known experimental chemical shifts δ, using trimethylsilane (TMS) as reference. The corresponding calibration curves for ^1^H and ^13^C chemical shifts are shown in Figures S3 and S4.

### 4.4. Antioxidant Activity Assays

The scavenging activity on the stable free radical DPPH was assayed by the modified Blois’ method in which the bleaching percentage of DPPH is monitored at a characteristic wavelength in the presence of the sample [57]. RA and CA stock solutions were prepared in DMSO. Calcium chloride stock solution was prepared in 1mM (NH_4_)2(CO_3_) buffer (pH 7.8). The assay was performed as follows: 100 μL of RA or CA solution (concentration 50 µM) in ammonium carbonate pH 7.8 aged for 1 h were mixed with 400 μL of TRIS buffer pH 7.4 and 500 μL of a fresh solution of DPPH at 100 μM in ethanol. Absorbance was measured at 517 nm after 20 min of reaction in the dark. DPPH inhibition was calculated by the equation:

%inhibition = [1 2212 (A_sample_/A_DPPH solution_)] × 100 (1)

where A_sample_ is the absorbance of the sample and A_DPPH solution_ is the absorbance of the DPPH solution. For calcium effect testing, an RA:Ca^2+^ or CA:Ca^2+^ ratio of 1:10 was used, and solutions of RA or CA with calcium was prepared 1 h before the assay.

## Figures and Tables

**Table 1:** Minimal energies of selected configurations for 1:1 complexes of di-deprotonated CA and RA with one Ca^2+^ ion.

Configuration	Deprotonation Site(s)	Complexation Site(s)	Energy (eV)
Chlorogenic acid			
**CAI**	C8–C20	C8	−266.07
**CAII**		C19–C20	−265.12
Rosmarinic acid			
**RAI**	C14–C3	C14	−270.97
**RAII**		C3–C4	−268.86
**RAIII**	C14–C20	C14	−269.29
**RAIV**		C19–C20	−268.58

## Supplementary Materials

Supplementary materials can be found at https://www.mdpi.com/1422-0067/21/14/4948/s1.

## Abbreviations

CA	Chlorogenic acid, (1S,3R,4R,5R)-3-{[(2E)-3-(3,4-dihydroxyphenyl)prop-2-enoyl]oxy}-1,4,5- trihydroxycyclohexanecarboxylic acid
DFT DPPH TRIS	Density functional theory 2,2-diphenyl-1-picrylhydrazyl tris(hydroxymethyl)aminomethane
RA	Rosmarinic acid, (2R)-3-(3,4-dihydroxyphenyl)-2-{[(2E)-3-(3,4-dihydroxyphenyl)prop-2-enoyl] oxy}propanoic acid
