# Supplementary material for: Interactions of Calcium with Chlorogenic and Rosmarinic Acids: An Experimental and Theoretical Approach
Source: Int J Mol Sci. 2020 Jul 13;21(14):4948. doi: 10.3390/ijms21144948 (PMC7403997; doi:10.3390/ijms21144948)
Supplement: Supplementary file 1 [file ijms-21-04948-s001.pdf]

Interactions of calcium with chlorogenic and rosmarinic acids: an experimental and theoretical approach

Estelle Palierse, Cédric Przybylski, Dalil Brouri, Claude Jolivalt, Thibaud Coradin

Supplementary Material

Figure **S1**: Experimental ^{1}H and ^{13}C chemical shifts of chlorogenic acid (a) in the absence and (b) in the presence of calcium (CA:Ca = 0.1) in D_2O and their attribution.

Figure **S2**: Experimental ^{1}H and ^{13}C chemical shifts of rosmarinic acid (a) in the absence and (b) in the presence of calcium (RA:Ca = 0.1) in D_2O and their attribution

Figure **S3**: Correlation between experimental chemical shifts (δ) and corresponding calculated absolute isotropic shielding σ X-AXIS for ^{1}H in selected molecules

Figure **S4**: Correlation between experimental chemical shifts (δ) and corresponding calculated absolute isotropic shielding σ X-AXIS for ^{13}C in selected molecules

Figure **S5:** Comparison between calculated (x-axis) and experimental (y-axis) chemical shifts for chlorogenic acid. (a)^{1}H chemical shifts, (b) ^{13}C chemical shifts

Figure **S6**: Comparison between calculated (x-axis) and experimental (y-axis) chemical shifts for rosmarinic acid. (a)^{1}H chemical shifts, (b) ^{13}C chemical shifts

Table **S1**: Experimental and predicted ^{1}H and ^{13}C chemical shift modifications of chlorogenic acid between CA alone and CA in the presence of calcium ion for configuration **CI**.

Table **S2**: Experimental and predicted ^{1}H and ^{13}C chemical shift modifications of chlorogenic acid between CA alone and CA in the presence of calcium ion for configuration **CI**

Table **S3**: Experimental and predicted ^{1}H and ^{13}C chemical shift modifications of rosmarinic acid between RA alone and RA in the presence of calcium ion for configuration **RI.**

Table **S4**: Experimental and predicted ^{1}H and ^{13}C chemical shift modifications of rosmarinic acid between RA alone and RA in the presence of calcium ion for configuration **RII**

Table **S5**: Experimental and predicted ^{1}H and ^{13}C chemical shift modifications of rosmarinic acid between RA alone and RA in the presence of calcium ion for configuration **RIII**.

Table **S6**: Experimental and predicted ^{1}H and ^{13}C chemical shift modifications of rosmarinic acid between RA alone and RA in the presence of calcium ion for configuration **RIV**.

(a)

C	δ (ppm)	H	δ (ppm)
5	37.29	5	2.04
3	38.39	3	
2	70.71	1	3.81
6	71.12	6	4.18
1	72.9	2	5.26
4	76.84	15	6.33
15	114.65	21	6.88
18	115.02	22	7.05
21	116.23	18	7.13
22	122.66	16	7.59
17	126.87		
19	144.34		
16	146.04		
20	147.30		
14	169.12		
8	180.88		

(b)

C	δ (ppm)	H	δ (ppm)
5	36.92	5	2.04
3	38.56	3	
2	70.82	1	3.83
6	70.87	6	4.27
1	72.63	2	5.25
4	77.92	15	6.28
15	113.85	21	6.82
18	114.61	22	7.06
21	116.23	18	7.12
22	122.87	16	7.55
17	125.84		
19	-		
16	146.45		
20	-		
14	169.28		
8	-		

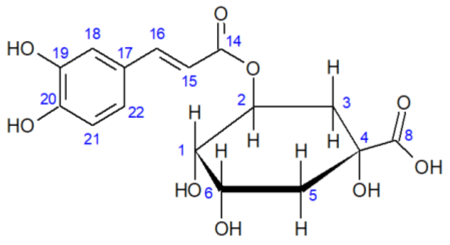

Figure S1: Experimental ^{1}H and ^{13}C chemical shifts of chlorogenic acid (a) in the absence and (b) in the presence of calcium (CA:Ca = 0.1) in D_2O and their attribution

(a)

C	δ (ppm)	H	δ (ppm)
7	36.74	7	2.92
8	76.30	7	3.04
18	114.43	8	4.97
15	115.08	15	6.22
21	116.12	5	6.72
5	116.12	6	6.77
2	117.16	2	6.8
6	121.73	21	6.82
22	122.63	22	6.94
17	126.99	18	7.04
1	130.17	16	7.42
4	142.57		
3	143.71		
19	144.16		
16	145.98		
20	146.96		
10	168.88		
14	177.04		

(b)

C	δ (ppm)	H	δ (ppm)
7	36.74	7	2.92
8	76.35	7	3.04
18	113.98	8	4.96
15	114.84	15	6.18
21	116.11	5	6.72
5	116.19	2	6.8
2	117.16	6	6.78
6	121.75	21	6.82
22	122.65	22	6.91
17	126.42	18	7.01
1	130.15	16	7.40
4	142.62		
3	143.76		
19	144.56		
16	146.17		
20	-		
10	169.01		
14	177.27		

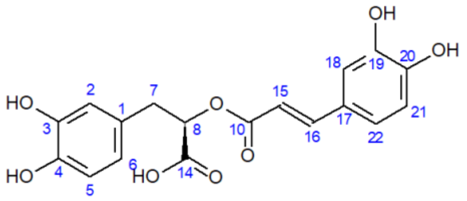

Figure **S2**: Experimental ^{1}H and ^{13}C chemical shifts of rosmarinic acid (a) in the absence and (b) in the presence of calcium (RA:Ca = 0.1) in D2O and their attribution

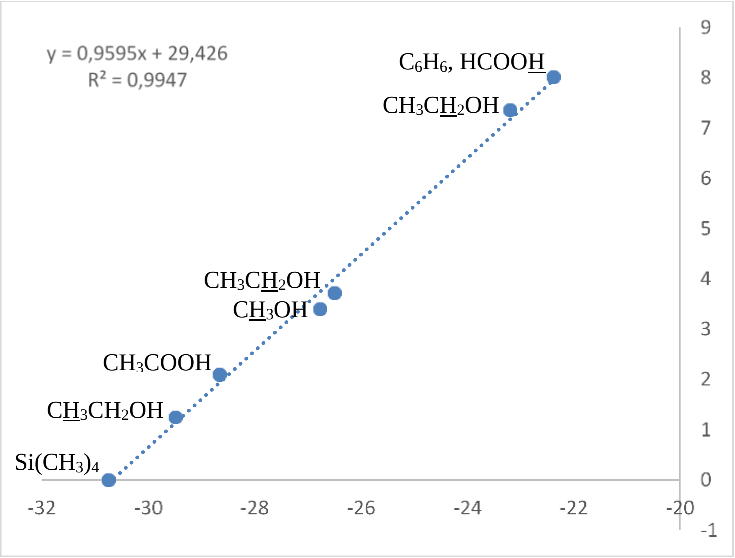

Figure **S3**: Correlation between experimental chemical shifts (δ) and corresponding calculated absolute isotropic shielding σ X-AXIS for $_1$H in selected molecules (In case of more than one kind of H atoms in the same molecule, the atom concerned is underlined): $Si(CH_3)_4$, CH_3CH_2OH, CH_3COOH, CH_3OH, CH_3CH_2OH, C_6H_6, HCOOH

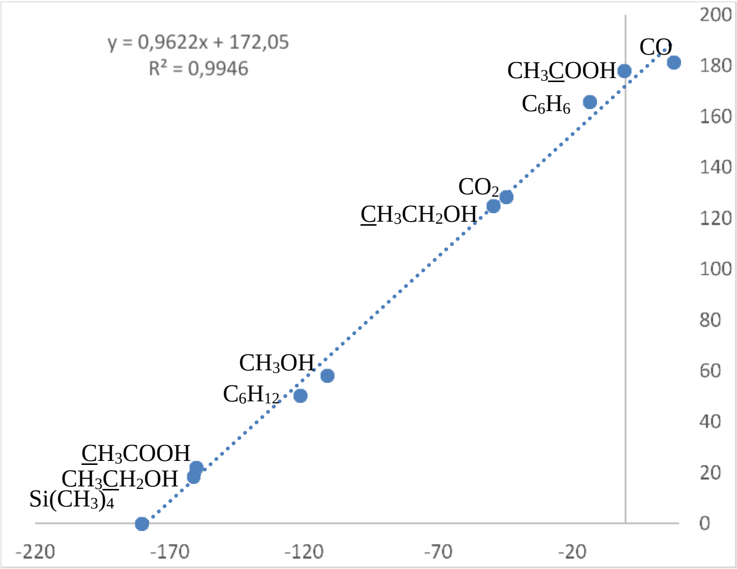

Figure **S4**: Correlation between experimental chemical shifts (δ) and corresponding calculated absolute isotropic shielding σ X-AXIS for $_{13}$C in selected molecules (In case of more than one kind of C atoms in the same molecule, the atom concerned is underlined): $Si(CH_3)_4$, $CH_3\underline{C}H_2OH$, $\underline{C}H_3COOH$, C_6H_{12}, CH_3OH, $\underline{C}H_3CH_2OH$, CO_2, C_6H_6, $CH_3\underline{C}OOH$, CO

(a) (b)

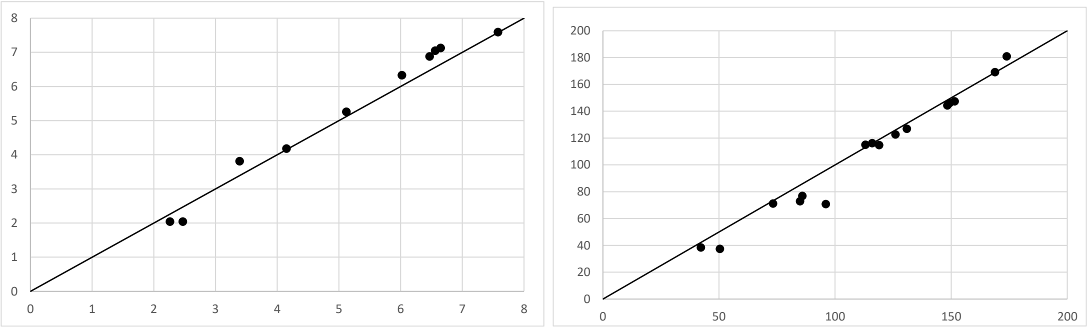

Figure **S5**: Comparison between calculated (x-axis) and experimental (y-axis) chemical shifts for chlorogenic acid. (a)^{1}H chemical shifts b) ^{13}C chemical shifts

(a) (b)

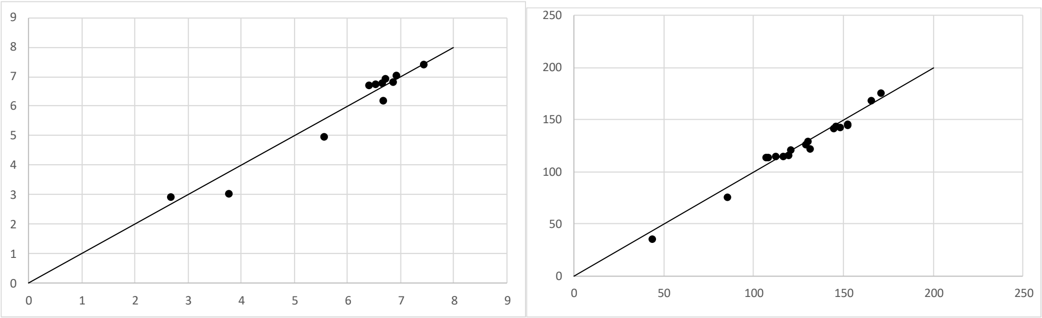

Figure **S6**: Comparison between calculated (x-axis) and experimental (y-axis) chemical shifts for rosmarinic acid. (a)^{1}H chemical shifts, (b) ^{13}C chemical shifts

^{1}H	Calculated Δδ (CA alone–complex)	Experimental Δδ (CA alone–complex)
5	-0.52	nd
5	-0.14	
3	-0.09	
3	0.03	
1	0.03	-0.02
6	0.43	-0.09
2	-0.05	0.01
15	0.68	0.05
21	0.82	0.06
22	0.09	-0.01
18	-0.04	0.01
16	0.77	0.04

^{13}C	Calculated Δδ (CA alone – complex)	Experimental Δδ (CA alone–complex)
5	3.74	0.37
3	-0.25	-0.17
2	8.05	-0.11
6	0.38	0.25
1	3.28	0.27
4	-23.67	-1.06
15	26.74	0.8
18	5.2	0.40
21	-6.59	0
22	-4.07	-0.21
17	-0.54	1.03
19	2.57	
16	15.15	-0.41
20	-28.52	
14	6.03	-0.16
8	-0.31	

Table **S1**: Experimental and predicted ^{1}H and ^{13}C chemical shift modifications of chlorogenic acid between CA alone and CA in the presence of calcium ion for configuration **CI**. In red, experimental variations >0.4 ppm for ^{13}C and >0.04 ppm for ^{1}H. In blue, calculated variations >0.4 ppm for ^{1}H and >10 ppm for ^{13}C

^{1}H	Calculated Δδ (CA alone–complex)	Experimental Δδ (CA alone–complex)
5	-0.42	nd
5	-0.13	
3	0.17	
3	-0.43	
1	-0.19	-0.02
6	0.55	-0.09
2	-0.16	0.01
15	0.09	0.05
21	-0.08	0.06
22	-0.38	-0.01
18	-0.31	0.01
16	-0.04	0.04

^{13}C	Calculated Δδ (CA alone –complex)	Experimental Δδ (CA alone–complex)
5	4.34	0.37
3	-2.02	-0.17
2	-0.03	-0.11
6	-0.79	0.25
1	1.82	0.27
4	2.76	-1.06
15	1.52	0.8
18	6.13	0.40
21	-3.16	0
22	-8.72	-0.21
17	-2.16	1.03
19	-1.06	
16	1.35	-0.41
20	-3.09	
14	-1.16	-0.16
8	-5.32	

Table **S2**: Experimental and predicted ^{1}H and ^{13}C chemical shift modifications of chlorogenic acid between CA alone and CA in the presence of calcium ion for configuration **CII**.

^{1}H	Calculated Δδ (RA alone-complex)	Experimental Δδ (RA alone-complex)
7	-0.16	0
7	0.23	0
8	-0.19	0.01
15	0.86	0.04
5	0.16	0
2	0.41	-0.01
6	-0.15	0
21	0.36	0
22	-0.12	0.03
18	0.46	0.03
16	0.07	0.01

^{13}C	Calculated Δδ (RA alone-complex)	Experimental Δδ (RA alone-complex)
7	-0.58	0
8	3.79	-0.05
18	2.48	0.45
15	19.09	0.24
21	-9.47	0.01
5	1.98	-0.07
2	-2.3	0
6	4.4	-0.02
22	-4.29	-0.02
17	10.47	0.57
1	-2.52	0.02
4	0.03	-0.05
3	-0.96	-0.05
19	-6.08	-0.4
16	4.2	-0.19
20	-18.11	
10	0.93	-0.19
14	-26.44	-0.23

Table **S3**: Experimental and predicted $_{1}H$ and $_{13}C$ chemical shift modifications of rosmarinic acid between RA alone and RA in the presence of calcium ion for configuration **RI.**

^{1}H	Calculated Δδ (RA alone-complex)	Experimental Δδ (RA alone-complex)
7	0.23	0
7	-0.27	0
8	0.09	0.01
15	-0.26	0.04
5	-0.05	0
2	-0.78	-0.01
6	0.32	0
21	0.11	0
22	0.12	0.03
18	-0.05	0.03
16	0.25	0.01

^{13}C	Calculated Δδ (RA alone-complex)	Experimental Δδ (RA alone-complex)
7	-1.02	0
8	0.53	-0.05
18	-0.2	0.45
15	-7.2	0.24
21	1.33	0.01
5	2.44	-0.07
2	-2.67	0
6	-5.07	-0.02
22	2.27	-0.02
17	-2.02	0.57
1	-14.84	0.02
4	-2.35	-0.05
3	-1.12	-0.05
19	0.38	-0.4
16	5.56	-0.19
20	2.43	
10	-1.39	-0.19
14	-0.43	-0.23

Table **S4**: Experimental and predicted $_{1}$H and $_{13}$C chemical shift modifications of rosmarinic acid between RA alone and RA in the presence of calcium ion for configuration **RII.**

^{1}H	Calculated Δδ (RA alone-complex)	Experimental Δδ (RA alone-complex)
7	-0.55	0
7	0.54	0
8	-0.25	0.01
15	-0.13	0.04
5	-0.11	0
2	1.06	-0.01
6	-0.69	0
21	0.04	0
22	0.06	0.03
18	0.07	0.03
16	-0.05	0.01

^{13}C	Calculated Δδ (RA alone-complex)	Experimental Δδ (RA alone-complex)
7	-5.08	0
8	1.04	-0.05
18	0.39	0.45
15	1.67	0.24
21	0.32	0.01
5	0.46	-0.07
2	-15.09	0
6	14.2	-0.02
22	0.55	-0.02
17	-0.63	0.57
1	-10.5	0.02
4	-9.98	-0.05
3	-13.71	-0.05
19	0.29	-0.4
16	0.79	-0.19
20	0.94	
10	2.78	-0.19
14	-16.66	-0.23

Table **S5**: Experimental and predicted ^{1}H and ^{13}C chemical shift modifications of rosmarinic acid between RA alone and RA in the presence of calcium ion for configuration **RIII.**

^{1}H	Calculated Δδ (RA alone-complex)	Experimental Δδ (RA alone-complex)
7	0.04	0
7	-0.29	0
8	0.03	0.01
15	-0.45	0.04
5	0.01	0
2	-0.58	-0.01
6	-0.04	0
21	0.17	0
22	0.22	0.03
18	-0.56	0.03
16	0.2	0.01

^{13}C	Calculated Δδ (RA alone-complex)	Experimental Δδ (RA alone-complex)
7	0.04	0
8	1.19	-0.05
18	-2.94	0.45
15	-10.26	0.24
21	-42.98	0.01
5	0.36	-0.07
2	-0.59	0
6	-3	-0.02
22	6.07	-0.02
17	-4.86	0.57
1	-5.43	0.02
4	2.17	-0.05
3	1.96	-0.05
19	-3.5	-0.4
16	6.27	-0.19
20	33.2	
10	-0.86	-0.13
14	-3.88	-0.23

Table **S6**: Experimental and predicted ^{1}H and ^{13}C chemical shift modifications of rosmarinic acid between RA alone and RA in the presence of calcium ion for configuration **RIV**. In red, experimental variations >0.4 ppm for ^{13}C and >0.04 ppm for ^{1}H. In blue, calculated variations >0.4 ppm for ^{1}H and >10 ppm for ^{13}C
